# Perceived consequences of healthcare service decentralization on access, affordability and quality of care in Khartoum locality, Sudan

**DOI:** 10.1186/s12913-021-06479-0

**Published:** 2021-06-17

**Authors:** Bandar Noory, Sara Hassanain, Jeffrey Edwards, Benedikte V. Lindskog

**Affiliations:** 1 The Epidemiological Laboratory, Department of Health System and Policy (HSP), Khartoum, Sudan; 2 International Community Health, University of Oslo, Oslo, Norway; 3 Federal Ministry of Health. Sudan, Khartoum, Sudan; 4 Department of Global Health, University of Washington, Seattle, Washington USA; 5 Department of International Studies and Interpreting, Section for Diversity Studies, OsloMet – Oslo Metropolitan University, Oslo, Norway

**Keywords:** Devolution, Implementation, Access, Affordability of health services, Quality of health services, Stakeholders

## Abstract

**Background:**

Decentralization of healthcare services has been widely utilized, especially in developing countries, to improve the performance of healthcare systems by increasing the access and efficiency of service delivery. Experiences have been variable secondary to disparities in financial and human resources, system capacity and community engagement. Sudan is no exception and understanding the perceived effect of decentralization on access, affordability, and quality of care among stakeholders is crucial.

**Methods:**

This was a mixed method, cross-sectional, explorative study that involved 418 household members among catchment areas and 40 healthcare providers of Ibrahim Malik Hospital (IBMH) and Khartoum Teaching Hospital (KTH). Data was collected through a structured survey and in-depth interviews from July–December 2015.

**Results:**

Access, affordability and quality of healthcare services were all perceived as worse, compared to before decentralization was implemented. Reported affordability was found to be 53 and 55% before decentralization compared to 24 to 16% after decentralization, within KTH and IBMH catchment areas respectively, (*p* = 0.01). The quality of healthcare services was reported to have declined from 47 and 38% before decentralization to 38 and 28% after, in KTH and IBMH respectively (*p* = 0.02). Accessibility was found to be more limited, with services being accessible before decentralization approximately 59 and 52% of the time, compared to 41 and 30% after, in KTH and IBMH catchment areas respectively, (*p* = 0.01).

Accessibility to healthcare was reported to have decreased secondary to facility closures, reverse transference of services, and low capacity of devolved facilities. Lastly, privatized services were reported as strengthened in response to this decentralization of healthcare.

**Conclusions:**

The deterioration of access, affordability and quality of health services was experienced as the predominant perception among stakeholders after decentralization implementation. Our study results suggest there is an urgent need for a review of the current healthcare policies, structure and management within Sudan in order to provide evidence and insights regarding the impact of decentralization.

**Supplementary Information:**

The online version contains supplementary material available at 10.1186/s12913-021-06479-0.

## Background

Since the 1990s there has been a substantial increase in the number of countries implementing health system decentralization [1]. Decentralization has become an integral component of health system reform packages focusing upon improving access, efficiency and the quality of care [2]. Enforcing governance and responsibilities at local levels has also been intentional goals of decentralization [2].

Decentralization has enhanced access to a broader range of health services closer to local communities [3–5]. It has also been associated with improvements in patient-provider relationships and community ownership due to the increased proximity [6]. Nevertheless, in many countries decentralization implementation and scale-up have faced challenges related to availability, affordability, and the quality of services. Studies have reported no or limited improvement with the quality of care after decentralization [3, 5]. Additionally, the specific effects of decentralization on the performance of health systems are poorly understood [1, 2, 7].

Sudan has been through several decentralization attempts. In 1994, federal decentralization at higher levels was implemented as part of broader economic and political liberalization policies. The consequence was the devolving of federal authorization to intermediate and local levels. However, it was reported that the devolution failed secondary to challenges during policymaking, including: policy formulation, implementation and evaluation [8]. The failure was blamed on political management, lack of community involvement, insufficient financial and human resources, and a lack of prioritization of the public health sector [8].

In 2011, decentralization with full transference of political, administrative, technical, and financial responsibilities was enacted where the responsibility of secondary and tertiary health facilities was transferred from the federal to state level. Additionally, rural hospitals and primary care facilities transferred similar responsibilities from the state level to local management. As a result of this policy change in 2012, healthcare services were shifted from large federal tertiary level hospitals to smaller district secondary hospitals.

This study focuses on the Khartoum locality, which faced decentralization with full transference of health services (administrative, technical, financial and political) from the federal tertiary Khartoum Teaching Hospital (KTH) located in center of Khartoum, to the district hospital Ibrahim Malik (IBMH), located in the southern part of Khartoum. The shift in health service availability before and after implementation of the decentralization policy of 2011 for the Khartoum locality, specifically KTH and IBMH are shown in Table 1.

**Table 1:** Availability of health services at KTH and IBMH before and after decentralization

Available services	Before decentralization		After decentralization	
	KTH	IBMH	KTH	IBMH
Emergency services				
General surgery	All services were available	Types of emergency services that were available:	Types of emergency services that were available:	Types of emergency services that available:
Orthopedics				
Urology		General surgery	General surgery	General surgery
Medicine		Medicine	Medicine	Colorectal surgery
Psychiatric		Obstetrics	Obstetrics	Medicine
Obstetrics		Gynecology	Gynecology	Obstetrics
Gynecology		Pediatrics	Pediatrics	Gynecology
Oncology				Pediatrics
Nephrology				Endocrinology
Dialysis				
Laboratory				
Radiology				
Surgery services		General surgery	N/A	General surgery
General surgery	Available			Colorectal
Colorectal				
Orthopedics				
Urology				
Internal medicine & subspecialties		Internal medicine	N/A	Internal Medicine
Gastroenterology	Available			Endocrinology
Cardiology				
Nephrology				
Endocrinology				
Neurology				
Rheumatology				
Oncology				
Pulmonology				
Obstetrics/Gynecology	Available	Available	N/A	Available
Pediatrics	NA	Available	N/A	Available
Psychiatry	Available	NA	NA	NA
Urology services	Available	NA	NA	NA
Pediatric surgery	Available	NA	NA	NA
Physiotherapy services	Available	NA	NA	NA
Endoscopy services	Available	NA	NA	NA
Nutritionist service	Available	NA	NA	NA
Isolation services	Available	NA	NA	NA
Laboratory and blood bank	Available	Laboratory services and blood bank	blood bank	Laboratory services and blood bank
Radiology services X-ray/ultrasound/CT	X-ray Ultrasound	X-ray	NA	X-ray
Nephrology & dialysis	Available	NA	NA	NA
Plastic surgery services	Available	NA	NA	NA
Orthopedics services	Available	NA	NA	NA
Neurology services	Available	NA	NA	NA
Intensive care unit (ICU)	ICU with 18 bed capacity	NA	NA	ICU with 7 bed capacity
Newborn nursey	Available	NA	NA	Available

*NA Not available * Available services: services that should be available in KTH and IBMH

There are no published studies in Sudan that we are aware of, about the perceived effect of decentralization on health service access, particularly at the community level. Utilizing a patient centered, conceptual framework for access, this study assessed the perceived effect of decentralization on access through a mixed methodology approach, focusing on availability of quality services and affordability as perceived by affiliated healthcare workers and people in local communities within the Ibrahim Malik and Khartoum Teaching hospitals catchment areas. The aim of our study was to determine the perceived effect of the transfer of health services from KTH to IBMH, looking at health service access after the implementation of the new decentralization policy.

## Methods

### Study design

This was a mixed-method study involving a cross-sectional household survey and qualitative explorative component, conducted from July through December 2015.

### General setting

Sudan is a low middle-income country with a population of approximately 37 million. The inhabitants are divided between rural (67%) and urban areas (33%) [9]. Administratively, Sudan is divided into 18 states. Poverty is widespread and the country’s debt was estimated at 42 billion USD in 2012 [9].

The health system is structured in three levels: 1) Federal Ministry of Health (FMOH), which is accountable for national planning; 2) State ministries, which are responsible for budget allocation and planning as well as administration of tertiary and secondary health service delivery; and 3) local health systems responsible for service delivery, organized by health regions [10, 11, 12].

### Specific setting

The Khartoum locality is part of the Khartoum state in Sudan and is the political capital of Sudan. It occupies about 176 km^2^, having a population of approximately 661,617 and includes 186 villages and 183 public administrative units (PAUs). It is divided into three administrative units (Khartoum, Alshohada, and Khartoum East); composed of urban, suburban, rural and internally displaced persons (IDPs) populations.

Khartoum has 31 public health care (PHCs) facilities, of which, 15 are referral health centers and 16 are primary healthcare units. In addition, there are 14 secondary and tertiary hospitals, 10 non-governmental organization (NGO) health centers, and 60 private health facilities. Decentralization with full transference of responsibilities was implemented in 2011 within the Khartoum locality. Subsequently, the Khartoum Teaching Hospital (KTH) healthcare workers and service delivery responsibilities were transferred to Ibrahim Malik district hospital (IBMH).

KTH is located in the northern area of Khartoum and before decentralization it was delivering secondary and tertiary healthcare services with an approximate 1100 bed capacity in Table 1. Following decentralization, KTH had less than 100 beds and received patients from all states of Sudan. IBMH is located in the southern part of the Khartoum locality. It was used to provide secondary healthcare services with approximately 100–150 beds available before decentralization and increased to approximately 280 beds following decentralization in Table 1. Some services have been transferred to other hospitals, such as nephrology and dialysis services to Bahri, and Alacademy hospitals. Whereas the majority of services have such been neglected and not transferred to any site including psychiatric services, isolation, clinical nutrition, plastic surgery, urology, physiotherapy and, neurological services. In addition, even the transferred services have not been done at the same equivalent capacity as it used, they had been previously to be in KTH due secondary to the lower capacity of the building of IBMH, which was set originally designed to function as a district first referral hospital.

### Selection and description of study participants

The study population comprised the heads of households in the Khartoum locality, the catchment area of the KTH and IBMH, and all healthcare workers of both hospitals.

### Study sampling

#### Quantitative component

For the quantitative part, the cross-sectional community members/household heads were selected randomly as per below mentioned stages. The participants were recruited through a household visit, after been informed about the study and the questionnaire.

The reported population of Khartoum locality is 661,617. A sample of 418 participants was calculated and then distributed to the locality based on numbers of households and population. A 95% confidence interval was utilized and a variance of 0.25 assuming that people in the target population have an equal chance to access health services (*p = 0.5*). The precision was assumed as 0.05 to reach the most accurate sample size. By using the population size of Khartoum locality (661,617) online formula calculator.net was used to calculate the sample size as 384 [13]. The sample size was adjusted to 418, after applying a 9% non-response rate.

The Khartoum locality was intentionally selected because decentralization has been implemented there to a greater extent. The locality was divided into formed clusters of 15 households per cluster. Three-stage, clustered, random sampling was utilized for recruitment of households at the community level where weighting of cluster, number of households in each cluster, and intervals of random selection were identified. Data collection, using a standardized questionnaire after piloting, occurred from July through December 2015.

Civil registration in Sudan is not 100% reliable; therefore participants were not selected via simple random sampling. Subsequently, three stages of stratified cluster random sampling, using an up to date sampling frame, were applied in the following manner:

##### Stage 1

The study targets within the household were the heads of the household (HH) or nearest relation. There were 418 households visited and the total number of HHs was distributed through the locality according to household weight within each cluster as described:

- Each cluster formed in the locality covered 15 households. Each locality was divided into the above-formed clusters as follows:
  - The weight of cluster (number of HHs in each cluster) = 15HHs
  - The total number of clusters = 418/15 = 28 clusters

##### Stage 2

Sample interval was determined as following:

$$ \mathrm{Sample}\ \mathrm{interval}=\mathrm{Total}\ \mathrm{number}\ \mathrm{of}\ \mathrm{cumulated}\ \mathrm{HHs}/\mathrm{total}\ \mathrm{number}\ \mathrm{of}\ \mathrm{clusters} $$

286,589

$$ \mathrm{Random}\ \mathrm{start}=\mathrm{first}\ \mathrm{start}+\mathrm{interval} $$

$$ \mathrm{First}\ \mathrm{start}=\mathrm{Interval}\ast \mathrm{RAND}\ \left(0-1\right)=\mathrm{13,297} $$

$$ \mathrm{Random}\ \mathrm{start}=\mathrm{19,522} $$

Then 28 popular administrative units (PAU) (clusters) were selected randomly by up to date sampling frame.

##### Stage 4

❖ To determine households that will be selected in each (PAU) (cluster), blocks or villages that constitute the area were selected firstly by random selection from the total number of blocks found in the PAU.

❖ Then HHS were selected by a simple random selection from the list of house numbers or list of family head names as following:

❖ A total number of houses or list of head of families/ 15 = sample interval.

❖ The total 15 HHs in each PAU were distributed in the selected block by the sample interval determined by the interval between selected household and the next one.

#### Qualitative component

Sampling for the qualitative component involved community members/household heads that were purposively sampled based on a sample framework of household heads generated from the quantitative community household survey. The household heads were the population of interest as per the study aim. The household heads were visited at their house similar to the quantitative study where they were informed about the study, among other ethical issues emphasized in the information sheets and written consent, the study objectives, voluntary participation and the right to withdraw from the study. The other population of interest was health care workers at KTH and IBHM hospitals. The health care workers were selected on basis of having worked longer than 5 years in the health care system in order to include experiences and knowledge about the process of implementation as well as perceptions of change after decentralization. The recruitment of health care workers for interviewing took place through visits to the hospitals, detailing information about the project, their rights as participants and the consent form. Time and place for the interview were adjusted to the health care workers’ work schedule and preference for place to be interviewed. Data collection for both groups continued until a deeper understanding and saturation was reached.

### Data collection and entry

#### Quantitative variables

The primary variables were the perceptions of stakeholders before and after decentralization implementation of health services (see Additional files 1, 2). Catchment area and demographic variables included: age, gender, education and means of livelihood and income. Households were questioned about availability of health facilities, availability of workforces and information, as well as needed medicines. The affordability of consultation, and medicines as well as quality of and access to services before and after decentralization were also collected. An English version of the questionnaire was translated into Arabic. It was then piloted and validated for clarity and consistency by introducing it to a representative sample size and modifications were done accordingly.

#### Qualitative component

A sample frame of the population of interest was demonstrated, in line with the study objectives of exploring the perceived effect of decentralization on access and quality of care. The frame for community members was based on the list of the quantitative part for household heads, residing in the KTH and IBHM catchment areas. Health worker selection was driven by workforce experience for both KTH and IBHM, including only those with at least 5 years’ experience, to be able to provide knowledge regarding the process of decentralization implementation and the perception of change after decentralization. Approximately 20 community members were purposively recruited through household visits and interviewed within a private area in the community and based on their preferences. Forty health workers were recruited from KTH and IBHM and interviewed in an agreed upon private area to try and limit biases. Recruitment of staff members was conducted through hospital visits. Interview time and place were based upon participants’ preferences.

The objective of the study was illustrated clearly through provided informational documents and written consents. Group homogeneity was assured, and community members included (7 females and 13 males, age range of 22–70 years). Health workers included 20 medical doctors, six nurses, four lab technicians, four pharmacists, two statisticians, and four administrative staff from KTH and IBHM (work experience range from 5 to 23 years).

Interview guides which were initially structured in alignment to study objectives, based on extensive literature review and contextualization (see Additional files 3, 4, 5, and 6). They were then piloted and pre-tested with ten community members and ten healthcare providers in catchment areas and a hospital in the Khartoum state that have a similar setting as the study site for clarity, coherence, and relevance, modifications were made in the topic guides accordingly. The duration of interviewing was approximately 60 min and utilized both recorded audio and field notes. The interviewer was a medical doctor, trained in qualitative research and had a similar cultural and lingual background as the study participants. Reflexivity has been ensured all through by means of cross-checking of data, daily debriefing and reviewing of audio-recordings with the research team, as well as on-going evaluation of the role of researchers in the interview process.

### Data analysis

#### Quantitative component

Chi-square testing was used for categorical variables to measure the perception of change in the access, affordability, quality, and availability of health services, availability of health staff, medications and health information before and after decentralization implementation and according to the catchment area of the study participants. The McNemar test was done to measure the perception of change in availability, affordability, accessibility, and quality of health services before and after decentralization. T–testing was used to compare continuous variables. Data were entered and analyzed using the Statistical Package for the Social Sciences software (SPSS) version 22 (IBM Corp, Armonk, NY, USA). The association between dependent and independent variables was measured by odds ratio (OR), utilizing 95% confidence intervals. Analysis at univariate and multivariate level by logistical regression was conducted, with a statistical association for *p*-values < 0.05.

#### Qualitative component

Qualitative data from the interviews were extracted. An inductive approach was systematically utilized in order to analyze and provide linkages between narratives and study objectives. The qualitative thematic content analysis was then completed by reviewing transcripts and then coding to identify key themes that were specifically relevant to the research questions. The qualitative thematic content analysis was done by reading through transcripts many times and then coded in order to identify key themes and sub-themes that emerged from the transcribed data material.

Four main thematic areas were identified:

1. Stakeholders’ perception of change in the *availability* of health services before/after decentralization.
2. Perception of change regarding the *affordability* of health services before/after decentralization.
3. The experienced change in the *quality* of health services before/after decentralization.
4. Intra-regional *inequalities* concerning the access to health services.

For this study, *access* to health services within the catchment area was defined as the ease in which communities or consumers can receive health services appropriate to their needs (1). *Availability* of health services was characterized by the physical existence of health resources with sufficient capability to deliver health services required at the time of the need (2). Lastly, *affordability* was interpreted as the capabilities of individuals to pay out resources and time to utilize the service. It is determined by the direct expense of service and related costs (3).

### Ethical consideration and informed consent

Ethical clearances were obtained from the Norwegian REK (Regional Committee for Medical-Health Research Ethics, reference number 2015/937/−REK) and from the Norwegian Social Science Data Services (reference number NSD-44106/3/LB) as well as from the Federal Ministry of Ethical Health Committee of Sudan. Written informed consent was obtained from all participants after reviewing a structured clarification of the research topic and objectives. Confidentiality and anonymity were preserved, and recruitment was voluntary after informing participants they had the right not to participate or to withdraw from the study without any consequences or any harm.

## Results

### Quantitative component

#### Socio-demographic profile of respondents

Among household respondents, there were 215 females (51%) and 203 males (49%). Approximately 68% of respondents were married and 26% were single in both the catchment areas of KTH and IBMH. For respondents who reported completing university studies or higher education, 39% were from the KTH catchment area while 48% were from the IBMH region, see Table 2. The mean income of study participants was 2245 Sudanese Pound (SDG). Low monthly income (based on minimum daily wages of Sudan) was reported by 4% of household heads within the KTH catchment compared to 5% in IBMH. Approximately 47% of households in KTH were composed of 5–10 members, while 55% of households in IBMH catchment area were of similar size. In both areas, the majority of household heads fell within an age range of 18–58 years.

**Table 2:** Sociodemographic profile of the head of households in catchment areas of KTH and me IBMH, Khartoum Locality Sudan, July – December 2015

Characteristics	Household in KTH area^a^ N (%)	Household in IBMH area^b^ N (%)	Total N (%)
**Education**			**418**
Illiterate	5(2%)	4 (2%)	9 (4%)
Non-formal	4 (2%)	10 (5%)	14 (7%)
Primary	25 (13%)	26 (12%)	51 (25%)
Intermediate	19 (10%)	15 (7%)	34 (17%)
Secondary	69 (34%)	58 (26%)	127 (60%)
University and above	77 (39%)	106 (48%)	183 (87%)
**Monthly income**			**418**
High	123 (62%)	141 (65%)	264 (64%)
Medium	68 (34%)	64 (30%)	132 (32%)
Low	7 (4%)	11 (5%)	18 (4%)
**Age group (years)**			**418**
18–38	87 (44%)	105 (48%)	192 (46%)
38–58	85 (43%)	83 (43%)	168 (40%)
58–78	26 (13%)	29 (13%)	55 (13%)
> 78	1 (1%)	2 (1%)	3 (1%)

^a^*KTH* Khartoum Teaching Hospital catchment area; ^b^*IBMH* Ibrahim Malik Teaching Hospital catchment area

### Availability, affordability, accessibility, and quality of health services

When measuring the perception of *availability* of health services across both catchment areas of KTH and IBMH, there were no significant changes before/after decentralization, Table 3. However, when comparing the change in perception of availability of health services before/after decentralization individually, both KTH (90% before vs 48% after, *p* < 0.0001) and IBMH (88% before vs 42% after, *p* < 0.0001) had significant decreases reported. Availability of health workers also decreased before/after decentralization comparing KTH (63% vs 48%, *p* = 0.002) and IBMH (60% vs 46%, *p* = 0.003) catchments. In addition, the perception of overall health service *affordability* decreased considerably for those in the KTH (53% vs 24%, *p* < 0.0001) and IBMH (55% vs 16%, *p* < 0.0001) areas. Despite a decrease in the availability and affordability of health services, availability of medications was reported before/after decentralization as increased in both KTH (31% vs 51%, *p* < 0.0001) and IBMH (31% vs 46%, *p* = 0.001) areas.

**Table 3:** Perceived effect of decentralization on *availability* and *affordability* by the head of households in catchment areas of Khartoum Teaching Hospital compared with Ibrahim Malik Hospital, Khartoum Locality Sudan, July to December 2015

Elements	Response	Household in KTH area^a^	Household in IBMH area^b^	***P*** value
**Availability of health services**	Yes	179 (90%)	192 (88%)	0.28
**Before decentralization**	No	20 (10%)	27 (12%)	
	Yes	95 (48%)	91 (42%)	0.12
**After decentralization**	No	104 (52%)	128 (58%)	
**Availability of health workers**	Yes	125 (63%)	106 (48%)	0.002
**Before decentralization**	No	74 (37%)	113 (51%)	
	Yes	119 (60%)	100 (46%)	0.003
**After decentralization**	No	80 (40%)	119 (54%)	
**Availability of medications**	Yes	61 (31%)	67 (31%)	0.54
**Before decentralization**	No	138 (69%)	152 (69%)	
	Yes	101 (51%)	101 (46%)	0.24
**After decentralization**	No	98 (49%)	118 (54%)	
**Availability of health information**	Yes	71 (36%)	70 (32%)	0.2
**Before decentralization**	No	128 (64%)	149 (68%)	
	Yes	85 (43%)	73 (33%)	0.03
**After decentralization**	No	114 (47%)	146 (67%)	
**Affordability of services**	Yes	105 (53%)	121 (55%)	0.34
**Before decentralization**	No	94 (47%)	98 (45%)	
	Yes	49 (24%)	36 (16%)	0.03
**After decentralization**	No	150 (75%)	183 (84%)	

^a^*KTH area* Khartoum Teaching Hospital catchment area; ^b^*IBMH area* Ibrahim Malik Teaching Hospital catchment area

Approximately half of the study participants (*n* = 205) expressed their perception of change in affordability after decentralization implementation. One hundred fifty nine participants experienced the perception of worsening affordability of health services and their perception remained after decentralization. Thirty three participants perceived change in the affordability of health services from unaffordable to affordable after decentralization. One hundred seventy two participants perceived change in the affordability of health services before/after decentralization to becoming unaffordable, while 52 perceived no change (*p* = 0.00).

Regarding accessibility to healthcare services, 168 participants reported a perception of change in accessibility to health services after decentralization (*p* = 0.00), Table 4. For 145 participants health services remained inaccessible before and after decentralization, while 41 participants perceived a change in the accessibility from inaccessible before decentralization to accessible after decentralization. Two hundred fifteen participants perceived the quality of health services had worsened following decentralization, while 25 participants perceived improvement in quality.

**Table 4:** Perceived effect of decentralization on the *access* and *quality of services* by head of households in catchment areas of Khartoum Teaching Hospital and Ibrahim Malik Hospitals, Khartoum Locality, July to December 2015

Elements	Response	Household in KTH area^a^	Household in IBMH area^b^	***P*** value
**Access to care**	Yes	118 (59%)	114 (52%)	0.082
**Before decentralization**	No	81 (41%)	105 (48%)	
	Yes	81 (41%)	65 (30%)	0.012
**After decentralization**	No	118 (59%)	154 (70%)	
**Quality of care**	Yes	94 (47%)	84 (38%)	0.041
**Before decentralization**	No	105 (53%)	135 (62%)	
	Yes	75 (38%)	61 (28%)	0.021
**After decentralization**	No	124 (62%)	158 (72%)	

^a^*KTH* Khartoum Teaching Hospital catchment area. ^b^*IBMH* Ibrahim Malik Teaching Hospital Catchment area

**Fig. 1:**
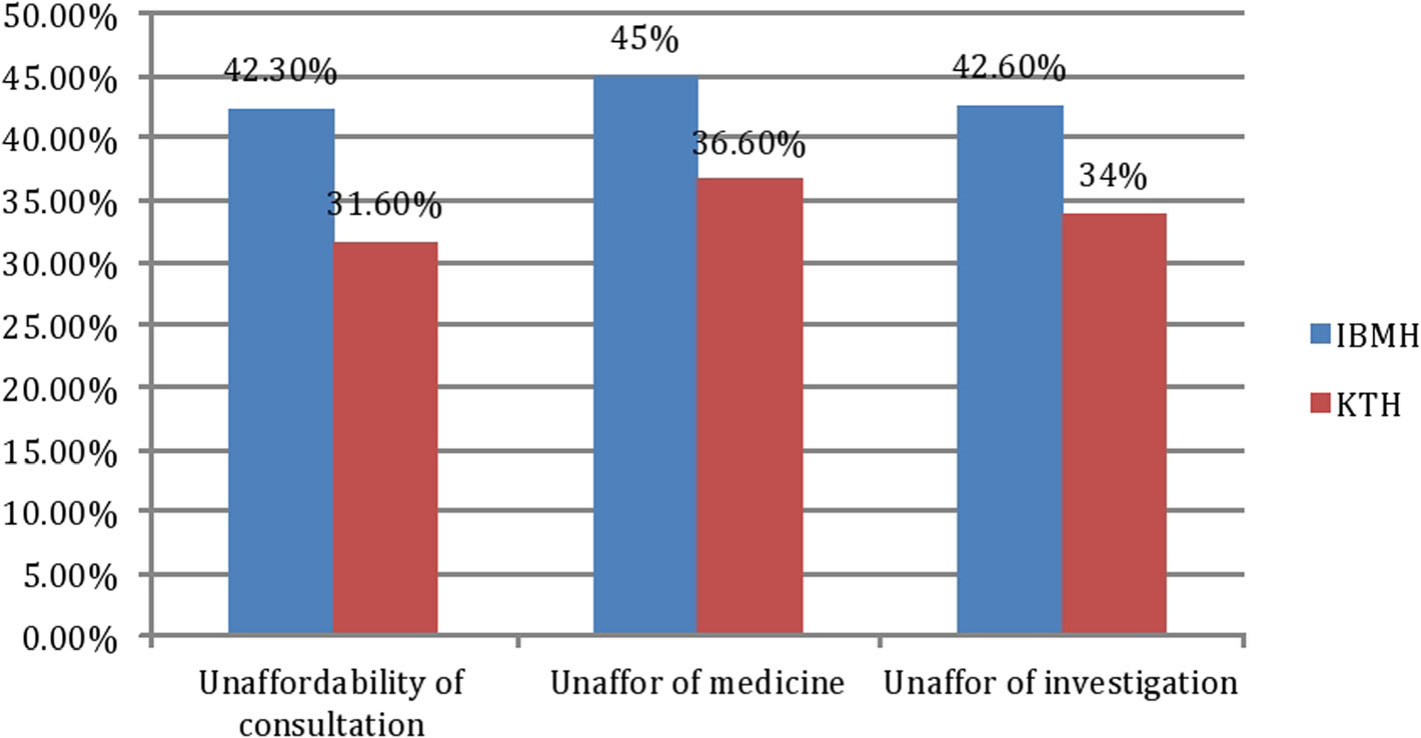
Proportion of people who percieved cost of consultation, investigation and medicines as unaffordable in Khartoum Teaching Hospital (KTH) and Ibrahim Malik Hospital (IBMH) in Khartoum Locality Sudan, July to December 2015

Consultation fees were reported as unaffordable after decentralization by 42% in the IBMH area compared to 31.6% of households within the KTH catchment, (*p* = 0.001), The proportion of people in the IBMH area who perceived the cost of medications and lab investigations as affordable before/after decentralization was 7.4 and 11% respectively (*p* = 0.01), compared to 9.8 and 13.6% (*p* = 0.01) among respondents from KTH area, (Fig. 1). There was an association between perceived unaffordability after decentralization with consultations (*p* = 0.001), medications (*p* = 0.01) and laboratory investigations (*p* = 0.01), in both KTH an IBMH catchment area. Payment for healthcare services via health insurance support after decentralization was reported by 71% of respondents in IBMH and 58% in KTH catchment areas (*p* = 0.004).

Access to care was reported to be perceived as decreased after decentralization by both those in KTH and IBMH catchment areas, see Table 4. Within each catchment area individually, there was a greater reported decrease in access to health care before/after decentralization, KTH was 59% before vs 41% after (*p* < 0.001) and IBMH was 52% vs 30% (*p* < 0.001). Likewise, a decline in the quality of healthcare services after decentralization was reported by 62% of respondents in the KTH and 72% in the IBMH catchment areas (*p* = 0.02).

### Determinant factors of affordability and availability

A univariate analysis revealed that payment for consultation, catchment area, access after decentralization, and type of payment were significant predictors of the perceived deterioration in affordability, see Table 5. The adjusted analysis model showed that individuals who paid > 100 Sudanese pounds (SDG) per facility visit and residing in the KTH area were 3.6 times more likely to report that affordability of a consultation had worsened. In comparison, those in IBMH area and who paid less than 100 SDG (OR 3.6, CI = 1.5–9.0, *p* = 0.005), were 5.9 times greater (OR 5.9, CI = 2.1–16.6, *p* = 0.001) to report worsening of affordability following decentralization. Individuals who did not have access to services in KTH were 4.5 times more likely (OR 4.5, CI = 2.1–9.8, *p* < 0.001) and in IBMH area, were 3.4 times likely (OR 3.4, CI = 1.8–6.4, *p* < 0.001) to state that affordability of consultation had declined after decentralization.

**Table 5:** Determinants of perceived affordability among households in catchment areas of Khartoum Teaching Hospital and Ibrahim Malik Hospitals, Khartoum Locality, Sudan, July to December 2015

Determinants	OR (95% CI)	***P*** value	OR (95% CI) adjusted	***P*** value
**Sex** (ref = male)				
**Female**	0.90 (0.55–1.48)	0.69	0.868 (0.514–1.465)	0.595
**Catchment area** (ref = KTH)				
**IBMH**	0.55 (0.33–0.91)	0.019	0.63 (0.30–1.30)	0.21
**Access after decentralization** (ref = no)	–		–	
**Yes**	5.47 (3.21–9.31)	0	4.54 (2.12–9.76)	0
**Payment for drug** (ref < 20 SDG)				
**20–50 SDG**	3.93 (1.90–8.12)	0		
**50–100 SDG**	1.23 (0.54–2.82)	0.63		
**more than 100 SDG**	7.57 (3.04–18.84)	0	3.64 (1.47–9.04)	0.005
**Income group** (ref = low)				
**Medium**	0.68 (0.07–6.63)	0.74	1.92 (0.62–5.98)	0.26
**High**	0.50 (0.03–7.54)	0.62		
**Annual cost** (ref < 1000)				
**1000–4999**			0.86 (0.47–1.56)	0.61
**5000–9999**				
**10,000–20,000**				
**> 20,000**				
**Type of payment** (ref = health insurance)				
**User fee**	1.97 (1.19–3.25)	0.008	1.44 (0.83–2.51)	0.2
**IBMH Catchment area**				
**Sex** (ref = male)				
**Female**	0.93 (0.59–1.45)	0.75	1.14 (0.61–2.12)	0.68
**Catchment area** (ref = IBMH)				
**KTH**	0.57 (0.36–0.91)	0.017	0.72 (0.38–1.36)	0.31
**Payment for investigation** (ref < 20 SDG)				
**20–50 SDG**	1.65 (0.70–3.90)	0.26	2.15 (0.75–6.18)	0.16
**50–100 SDG**	2.07 (0.82–5.18)	0.12	2.12 (0.72–6.23)	0.17
**> 100 SDG**	4.67 (2.07–10.53)	0	5.91 (2.10–16.58)	0.001
**Monthly income** (ref = low)				0.009
**Medium**	0.87 (0.09–8.48)	0.91	0.37 (0.03–4.72)	0.44
**High**	3.00 (0.25–36.33)	0.39	2.92 (0.18–46.88)	0.45
**Access after decentralization** (ref = no)	4.05 (2.52–6.51)	0	3.37 (1.79–6.36(	0
**Annual cost** (ref < 1000)				
**1000–4999 SDG**			1.77 (1.00, 3.13)	0.05
**5000–9999 SDG**	0.79 (0.08–7.80)	0.84	1.26 (0.11–14.18)	0.85
**10,000–20,000 SDG**	1.00 (0.10–10.54)	1	1.91 (0.16–23.06)	0.61
**> 20,000 SDG**	0.60 (0.05–7.92)	0.7	1.13 (0.08–16.8)	0.93
**Type of payment** (ref = health insurance**)**				
**User fee**	1.95 (1.23–3.09)	0.005	1.40 (0.70–2.80)	0.34
**Low income group KTH**				
**Sex** (ref = male)				
**Female**	0.95 (0.61–1.47)	0.81	0.98 (0.54–1.77)	0.94
**Catchment area** (ref = KTH)				
**IBMH**	0.47 (0.30–0.73)	0.001	0.67 (0.37–1.22)	0.19
**Payment for consultation** (ref < 20 SDG)				
**20–50 SDG**	1.88 (0.81–4.36)	0.14	2.56 (0.86–7.57)	0.09
**50–100 SDG**	1.30 (0.47–3.64)	0.62	2.14 (0.64–7.15)	0.22
**> 100 SDG**	4.05 (1.90–8.64)	0	4.54 (1.62–12.77)	0.004
**Income group** (ref = low)				
**Medium**	1.08 (0.11–10.51)	0.95	0.25 (0.02–3.17)	0.29
**High**	0.50 (0.03–7.54)	0.62	0.20 (0.008–5.05)	0.33
**Access after decentralization** (ref. = no)				
**Yes**	4.39 (2.77–6.97)	0	3.36 (1.81–6.25)	0
**Annual cost** (ref < 1000)				
**1000–4999**		0.999		0.999
**5000–9999**		0.999		0.999
**10,000–20,000**		0.999		0.999
**> 20,000**		0.999		0.999
**Type of payment** (ref = health insurance)				
**User fee**	2.44 (1.56–3.82)	0	1.37 (0.80–2.35)	0.25

#### Qualitative component

A total of 40 healthcare workers (21 male and 19 female) participated in in-depth interviews. Approximately 82% were between 25 and 50 years old. An additional 20 community members (13 male and 7 female) were also recruited from the household survey. Socio-demographic profiles of participants were collected at the beginning of each interview following consent. Approximately half of the healthcare workers were employed for at least 10 years in the hospitals. Among community members interviewed, 14% were unemployed, 36% were housewives, 45% had income-generating work and 6% were retired.

Several themes emerged from the interviews. To protect participants’ anonymity, quotes presented in this section are labeled with participants’ information code only.

##### Fragmentation and unsatisfying quality of services after implementation of decentralization

There were similarities in responses regarding the transfer of services from KTH to IBMH being perceived as a cause of fragmentation of services in both facilities. After decentralization, some services were not yet fully implemented within IBMH, which affected availability. The respondents perceived decentralization implementation as a barrier to availability and obtaining quality of care in an affordable manner.

Some study participants - both community members and healthcare providers - related that there was no need for the transfer of services from central hospitals to district facilities. Instead, many respondents emphasized that the service should be improved in the peripheries by the establishment of fully equipped facilities, while KTH should be improved to continue as a tertiary hospital. Physical and geographical inaccessibility around catchment areas was also reported as a negative impact of decentralization. “Why should I have to go to Ibrahim Malik while the nearest facility to my home is Khartoum Hospital? Where can I be treated now? In my area, there is only a poorly equipped healthcare center and many private facilities” (Interview 22 community member, Male. 19/10/2015). There is a ‘lack of emergency services as a consequence of the implementation process, as one healthcare provider, “Male” stated: “Transference of service lacked staging and proper coordination, which led to the denial of life-saving services to patients who came to KTH”.

Community members and healthcare providers stated that before decentralization there were comprehensive services in KTH, where a cluster of services and specialties were located. Therefore, if the patient came for treatment in one of the specialty departments and was recommended for surgery, the department of surgery and surgical subspecialties were available within the facility. Some community members reported that the available staff at IBMH was junior, with poor qualifications, and that senior staff relocated to the private sector or outside the country. Additionally, there was no regular availability of specialists in the decentralized IBMH hospital. Moreover, stopping the assignment of the resident, non-specialist doctors, affected the quality of services because service delivery became dependent on trainee doctors (house officers and deputy specialists) as stated by both community members and health care providers. A health worker stated, “In my opinion, everyone who gets sick is better off going to Khartoum hospital where he/she can find comprehensive service. Now after decentralization, they have stopped Khartoum hospital, except for the surgery department which will be shut down in the coming days “. (Interview 4. Specialist, Male 3/10/2015).

Some study participants described that healthcare staffs at peripheral facilities like IBHM are mainly junior doctors and house officers. They also mentioned that poorly qualified doctors usually serve at healthcare centers and peripheral hospitals. The participants attributed this to the out-flux and brain drain of qualified staff leaving, either to the private sector or overseas. “This presence of poorly qualified staff in the facilities led to the malpractice of abuse of investigation tools due to poor clinical skills” (interview 1. Community member. Female 2/10/2015). These changes were attributed as possible consequence of reform and the related to cut down in numbers of health workers, utilization of temporal contract and commoditization of training.

##### Unavailability of health services

Unavailability of health services was a central theme among study participants. They perceived that the decentralization process had decreased the availability of health services. This finding was supported by the household survey that reported a decrease in the availability of health services in both the KTH (90% before vs 48% after, *p* < 0.0001) and IBMH (88% before vs 42% after, *p* < 0.0001) catchment areas. Some mentioned that patients from the Khartoum state and other parts of Sudan lost the earlier well-established emergency services located at KTH, which were transferred to lower capacity and poorly equipped peripheral facilities. One healthcare worker reported: “There was a neonatology section which was also transferred and lost its position. It used to contain incubators and highly qualified nursing staff that was also shut down”. (Interview 43, Nurse. Female. 18/11/2015). Lack of preparation and lack of transfer of equipment during the process of decentralization were also mentioned by healthcare staff.

“The transference of service before the preparation of the peripheral facility led to inadequate services being delivered and unavailability of many types of healthcare services. In addition, due to inadequate investigation tools in the peripheral facilities, staff cannot deliver diagnostic services to patients and just do first aid and transfer patients to other facilities”. (Interview 43 Medical doctor. Male. 18/11/2015).

The transference of the medicine department from KTH to IBMH affected the delivery of trauma services, which are highly dependent upon consultative support and other medical departments as needed.

##### Unaffordability of health services

Participants considered costly and unavailable services, as a direct consequence of decentralization. User fee costs and the subsequent increase in consultation and medication charges were reported as significant barriers. These challenges were considered to be one of the primary barriers to affordability and consequently limiting equitable access to quality health services. Additionally, we found in the household survey that affordability and accessibility to health services was compromised after decentralization. Affordability was decreased in KTH from 53 to 24%, (*p* < 0.0001) and IBMH from 55 to 16% (*p* < 0.0001). Likewise, accessibility to healthcare services declined in KTH from 59% before to 41% after (*p* < 0.001) decentralization and in IBMH was 52% before and declined to 30% after (*p* < 0.001).

Participants specifically reported an increase in consultation service charges after decentralization from 17 SDG to 50 SDG (1.7–2 $), which some felt made services unaffordable in the district hospital despite being a public facility. There used to be a charitable collaborative treatment scheme to support vulnerable groups such as IDPs and those with low incomes. This, according to one community member had stopped after the implementation of decentralization (Interview 1, community member - Female. 2/10/2015).

Catastrophic cost was mentioned by community members as detrimental to seeking timely as well as good quality health care: “In this country, you may need to sell your house to cover hospitals’ bills” (Interview 21, Community member, Male. 17/10/2015). “Even the basic needs of gauze and syringe aren’t available and it’s us as citizens who buy them.” (Interview 8, Community member, Female. 7/10/2015).

##### Access and the change in health seeking behaviors

Non-medical costs related to the physical inaccessibility of services such as transportation cost may affect health seeking behavior in the sense that one may choose to seek care in a closer private clinic. Some of the study participants in the Khartoum area pointed out that after decentralization; the healthcare services became more geographically inaccessible. The Khartoum hospital was easier to access due to its strategic location at the center of the capital and in an area of the junction of different transportation lines. As noted by a community member “This added additional transportation costs as peripheral facilities like IBMH, are located in the Southern area and have no direct transportation lines, especially for those coming from other states of nearest regions” (Interview 1, Community member- Female. 2/10/2015). Another member expressed similar thoughts: “Hospitals services were transferred far from me and when considering transporting costs, I figured out that it’s better for me to seek care in private clinic. All this policy is generated in the favor of Al-Zaitona/a private hospital in the center of Khartoum Locality” (Interview 1 Community member- Female. 2/10/2015).

Self-healthcare also emerged as a topic of concern community members and healthcare providers stated that there had been an increase in seeking treatment and consultation directly from pharmacies and/or laboratories, due to high cost of treatment at health facilities. “People now go directly to pharmacies instead of clinic and take drugs due to high cost of healthcare”. (Interview 19 Pharmacist, Female. 15/10/2015).

The correlated dearth of some services has contributed to increasing its cost, as a participant mentioned that ICU and newborn services are also unaffordable in the public facilities due to shortages and cost 1500–2000 SDG (150–200$) a day.

Community members and health providers argued that indirect implications of unaffordability of medicines are serious, as it might double the burden on individuals, causing long delays before treatment and sometimes several self-treatment attempts that may lead to negative health outcomes. As noted by a community member; “After seeing a doctor, drugs are very expensive, more than 30 SDG. So, it took me 4-5 days to get money”. (Interview 2 Community member - Female. 2/10/2015).

“There is no free treatment at all; emergency drugs like ergometrine should be bought by the patient. Any patient buys his/her own medicine on his/her own money”. (Interview 59 Nurse, Female. 20/11/2015).

## Discussion

The key finding from this study was that collectively, there was an overall perception of decline in regard to access, affordability, and quality of health services after implementation of decentralization in Sudan. Interregional disparities of access have surfaced. In the KTH catchment area, the perceived decrease in accessibility could be attributed to transference of services away from the Khartoum area. Nonetheless, households surveyed in the IBMH area perceived more limitations in the access to healthcare services following decentralization, compared to those residing in the KTH area (*p* = 0.01, CI 0.41–0.92). These changes have contributed to alter negatively the prior comprehensiveness of services offered to people before decentralization. Our findings could be explained by variations in the managerial, financial and human resource capabilities between the KTH and IBMH hospitals [14]. However, a study within Sudan in 2003 also referred to the deterioration in health services after federal decentralization. It was found that the transference of authority and control of service provision to states and localities had weakened interrelated capacities [8]. The mechanism that was established to reduce gaps in the financial capabilities between states, which required national funding support, ended up widening the gap between intraregional inequalities in access to healthcare services [8]. The relationship between intraregional disparities in health services and the variation in the financial capabilities of the regions has been previously reported [15]. The negative consequences of decentralization have been also reported from Ghana and Tanzania [3, 5]. Similarly, an increase in intraregional disparities in access to health care services after decentralization has been found in studies from Canada, Switzerland, and China [15].

We found that the perceived unaffordability of services including clinical consultation, laboratory charges and medication expenses, can be a primary obstacle to healthcare accessibility and quality. This perceived unaffordability might be a reflection of the economic liberalization policy and is associated with implementation of structural adjustment programs in the health system more generally (i.e. - a regimen that imposes cost recovery schemes, user fees, reduction in health budgets and privatization of services) [16]. This national policy has caused a systematic weakening of the state and public sector, with the aim of promoting user fee and healthcare commodification (transformation of healthcare into objects of trades) by removing all types of public support [8]. Others have proposed that healthcare decentralization has been implemented with the aim of providing austerity-like programs, leading to withdrawal of the state from the provision of some health services and the subsequent introduction of user fees within the public sector [15].

Comparatively, fragmentation of healthcare services has emerged as one of the avoidable consequences of decentralization in the Philippines [1]. Decentralization in the Philippines led to a staff shortage secondary to reduced financial resources, and to frequent unavailability of medications, which was aggravated by the reluctance of some healthcare providers to work in peripheral areas [17]. Deficiencies in the quality of healthcare services provided after decentralization could be an inducing factor and driver for people to distrust and reject the public sector and seek services from private providers [18, 19].

Several participants reported having been subjected to catastrophic healthcare costs. Those residing in the IBMH catchment area were at 0.55 times higher risk to report consultation services as unaffordable, and 0.57 times more likely to imply that investigation services were unaffordable, compared to those residing within the KTH area.

Consequently, out-of-pocket expenditures for healthcare can increase vulnerability to households and exacerbate socio-economic risk. Lack of access to healthcare services, secondary to increased costs after decentralization, have been previously reported in Ghana, Uganda and Tanzania [3–5]. Moreover, this increases the risk of a poverty cycle for communities, given that there is a direct link between poverty, poor health outcomes and further poverty [20].

Deficiencies in meeting community needs in a comprehensive and affordable manner may lead to changing healthcare seeking behavior, including receiving care from unqualified providers, self-treatment and use of traditional healers. Our findings demonstrate challenges facing the achievement of universal healthcare access, coverage and quality care. Similar results have been reported in Nicaragua where universal healthcare coverage had been established, and after decentralization implementation, there was a 50% decline in prenatal and vaccination coverage [2]. These findings call for urgent action, especially in a setting like Sudan, where the inequality of resource distribution is the result of the long-term imposition of a capitalistic model of development [21].

Decentralized health services might also contribute to uphold these disparities or even serve to further widen the gap between those who can and cannot afford accessing appropriate and good quality care. Collectively, these effects may have significant developmental implications for the future state of health and healthcare service delivery in Sudan.

We believe that an updated analysis of healthcare policies and strategies in Sudan is needed, focusing on the effect of recent reforms, and ways to reduce this present perceived system fragmentation and to enhance possible achievable remedies for current gaps in care. In relation to the process of decentralization implementation, the Ministry of Health in Khartoum state held meetings that stipulated prior preparations of peripheral facilities by suitable equipment in agreement to decentralization decree. However, in reality the activities of the implementation overrode the agreement and the transference of services was disorganized, which also reflect discordances between the agreed-upon policy and actual practice.

We recommend that Sudan’s health strategy goals have an increased focus on reducing direct and indirect costs on local communities in an equitable manner. Reflected low accessibility and unaffordable services need to be urgently resolved within the public health system as well as with the lowest cost to prevent increased health risk and burden among households [19].

At a macro level, there is also a need for analyzing the financial theory related to the decentralization and its endorsement. Understanding compromises between policy decisions, decentralization advantages, and resulting consequences is essential. Demonstrating a solid framework that considers fully the implementation efficiency and cost at the community level is crucial [22].

### Strengths & limitations

The strength of this study is that it explores the effect of healthcare system decentralization as perceived by local communities as primary stakeholders, utilizing a mixed methods approach, which has not been previously conducted in Sudan. Use of qualitative methodology can enable one to capture multiple realities [23]. However, the subjective nature of interpretative analysis and the fact that the researcher is part of the analysis and interpretation, may affect the study findings [24]. Regardless, prolonged engagement with participants, triangulation of data sources and methodology were used to augment study results validity [23]. There are multiple complex factors that may be associated with the effects of decentralization on healthcare delivery and more research is needed to further understand the implications and relationship to long term health outcomes. Furthermore, before and after evaluation design can be affected by external factors. Hence, the changes that are presented in this study may not be due entirely to the effect of decentralization. This study is limited to the understanding of stakeholders’ perceptions about the change in access, quality, and affordability after decentralization implementation.

## Conclusion

The perception is that decentralization of services has likely contributed to reduced access, affordability and quality of healthcare in Sudan. Intraregional inequality of accessibility-imposed user fees and subsequent increased cost of care likely create direct and indirect burdens on households. Healthcare decentralization in Sudan thus far, is likely hindering the central goal of reducing public health risk, especially among the poor and vulnerable. Our findings call for future in-depth analyses of the Sudan healthcare system reforms, looking at key factors involved in the process of decentralization, its effect, and how they may impede health as a fundamental human right.

## Supplementary Information

**Additional file 1.** English version questionnaire:This file captures the perception of change among community members regarding the affordability, accessbility, availability and quality of healthcare services after decentralization implementation.

**Additional file 2.** Arabic version questionnaire.

**Additional file 3.** English version interview guides for community members:This file captues the experienced change in the availability, affordability, accessbility and quality of health care services after implementation of decentralization as experienced by community members.

**Additional file 4.** Arabic version of interview guides for community members: This file captues the experienced change in the availability, affordability, accessbility and quality of health care services after implementation of decentralization as experienced by community members.

**Additional file 5.** Arabic version of health care providers interview guides: This file captures the multiple realities regarding the perception of health care providers about the change in working environment, quality of delivered services after the implementation of decentralization.

**Additional file 6.** English version of health care providers interview guides: This file captures the multiple realities regarding the perception of health care providers about the change in working environment, quality of delivered services after the implementation of decentralization.

## Data Availability

The relevant data is included in the manuscript and the data set that support the conclusions of this article is available in the additional file.

## Abbreviations

IBMH: Ibrahim Malik hospital
KTH: Khartoum teaching hospital
USD: United States Dollar
FMOH: Federal ministry of health
PAUs: Public administrative units
HHS: Household
SDG: Sudanese pounds
OD: Odds ratio
REK: Regional Committee for Medical-Health Research Ethics
NSD: Norwegian Social Science Data Services
ICU: Intensive care unit
