# Supplementary material for: Perceived consequences of healthcare service decentralization on access, affordability and quality of care in Khartoum locality, Sudan
Source: BMC Health Serv Res. 2021 Jun 17;21:581. doi: 10.1186/s12913-021-06479-0 (PMC8212465; doi:10.1186/s12913-021-06479-0)
Supplement: Supplementary file 1 — Additional file 1. English version questionnaire:This file captures the perception of change among community members regarding the affordability, accessbility, availability and quality of healthcare services after decentralization implementation. [file 12913_2021_6479_MOESM1_ESM.pdf]

QUESTIONNAIRE

Questionnaire: Assessment of perceived effect of devolution on access to care and quality of care

Serial Number: ___

Personal Data

1- Sex:

1- Female () 2- Male ()

2-Marital Status

1- Single () 2- Married () 3- Divorced () 4- Widowed ()

3-Address: ____________________

4-Ethnic group: _______________

5-Education:

1- Illiterate ()
2- Non-formal ()
3- Primary ()
4- secondary ()
5- University and above ()

6-Age ……………………

7-Occupation: _____________

8-Monthly Income: ____________

Access to health care service? Availability of health facilities:

9-How many persons live in this household? ....

10-How many children living in this household are:(write the number in the box):

1- under 5 years old () 2- 5 years and older ()

11-Do you have health facility in your area?

1- Yes () 2-No ()

If yes,

12-What is the type of facility where you get your health care service?

1-Dressing station (DS) () 2-Dispensary ()

3-Health center (HC) () 4-Primary health care unit (PHCU) ()

5-Referral hospital (RH) () 6- Private sector (PS) ()

7- Go directly to pharmacy () 8- other ()

13-What is the distance of this facility from your home?

1-lessthan 5 Km () 2-5Km ( )

3-5 - 10Km ( ) 4- More than 10 Km ( )

14-Do you get health care service in facility in your area?

1-Yes ( ) 2-N0( )

15-Do you get the heath care service for free?

1- Yes ( ) 2- No ( )

If no,

16-What is the type of payment you have in the health facility?

1- User fee ( ) 2- health insurance ( )

17-Can you afford this payment?

1- Yes ( ) 2- No ( )

18-How much do you pay to get consultation service?

1- Less than 20 ( ) 2-20 – 50 SDG ( )

3-50 – 100SDG ( ) 4- more than 100 SDG ( )

19-How much do you pay for drugs?

1- Less than 20 ( ) 2-20 – 50 SDG ( )

2– 50 - 100SDG ( ) 4- more than 100 SDG ( )

20-How much do you pay for investigation?

1-Less than 20 ( ) 2-20 – 50 SDG ( )

3– 50 - 100SDG ( ) 4- more than 100 SDG ( )

21-How much do you buy for health service in a year ……………………

22-Do you have regular access to health service?

1- Yes ( ) 2- No ( )

If no,

23-What are factors that make your access to service irregular?

1- Health services not available ( ) 2- Financial constrains ( )

3-irregular availability of drugs ( ) 4- Long distance ( )

5- Irregular availability of health staff ( ) 6- lack of lab investigation ( )

7-Unqualified health staff ( ) 8-Lack of transportation ( )

9-Mal treatment by health staff ( ) 10-other ( )

24-Are health facilities open when you need them?

1- Yes ( ) 2-No ( )

25-If there is no health service in the nearby facility what do you do?

1-referred by health worker to other facility ( ) 2-Seek for other service ( )

3-Use home remedy ( ) 3-Go to traditional healer ( )

4- Get treatment from pharmacy ( )

26-Do the health service you get is appropriate to your needs?

1-Yes ( ) 2- No ( )

Quality of health care service:

27-Can you rank the quality of health service that you get after devolution?

1- Deteriorated ( ) 2-Improved

3- Improved ( ) 4-Do not know ( )

28-Do health workers regularly available in your health facility?

1- Yes ( ) 2-No ( )

If yes,

29-What health worker is available in your health facility?

1- Medical assistant ( ) 2- Nurses ( )

3-Medical Officer ( ) 4-Specialist ( )

5- Not specifies the health care provider ( )

30-Do drugs regularly available in your facility?

1-Yes ( ) No ( )

31-Do health related information available in your nearby facility?

1-Yes () 2-No ()

32-Do you trust on the health staff in the facility?

1-Yes () 2- No ()

33-Do waiting time for service in your facility is long?

1-Yes () 2-No ()

34- How much do you wait per minutes in your nearby facility?

…………………………..

35-Do you experience any improvement in access to service after devolution?

1-Yes ( ) 2-No ( )

36-Has the cost of treatment become affordable after devolution?

1-Yes ( ) () No

37-Do you feel welcomed in the health facility?

1-Yes ( ) 2- No ( )

38-Are satisfied with the quality of health service you get after devolution?

1-Yes ( ) 2-No ( )

39-What is the quality of buildings of health facilities?

1-Good ( ) 2-Bad ( )

40-What your perception about the change in quality of service indicators after devolution?

#	Criteria	Deteriorated	Improved
1	Availability of drugs		
2	Availability of health facilities		
3	Distance of facilities from home		
4	Affordability of consultation services		
5	Affordability of drugs		
6	Affordability of investigations		
7	Availability of health staff		
8	Quality of health facility		

	building		
9	Availability of health related information		
