# Supplementary material for: Perceived consequences of healthcare service decentralization on access, affordability and quality of care in Khartoum locality, Sudan
Source: BMC Health Serv Res. 2021 Jun 17;21:581. doi: 10.1186/s12913-021-06479-0 (PMC8212465; doi:10.1186/s12913-021-06479-0)
Supplement: Supplementary file 2 — Additional file 2. Arabic version questionnaire. [file 12913_2021_6479_MOESM2_ESM.pdf]

ARABIC QUSTIONNAIRE

استبيان: تقييم تأثير اللامركزية على الحصول على الرعاية وجودة الرعاية

الرقم المتسلسل:.....................

بيانات شخصية

1- الجنس:

أ- أنثى ( )

ب- ذكر ( )

2- التعليم:

أ- غير متعلم ( )

ب- تعليم غير نظامي( )

ت- الابتدائية ( )

ث- المتوسط ( )

ج- الثانوي ( )

ح- جامعى فما فوق ( )

3- الحالة الاجتماعية:

أ- أعزب ( )

ب- متزوج ( )

ت- مطلق ( )

ث- أرمل

4- العمر..........

5 - العنوان___________________

6 - المجموعه الإثنية_______________

7 - المهنة ____________

8 - الدخل الشهري___________

الوصول إلى خدمات الرعاية الصحية؟ توافر المرافق الصحية:

9- كم عدد أفراد هذه الأسرة؟ ....

10- كم عدد الأطفال الذين يعيشون في هذه الأسرة: (الرجاء كتابة الرقم في المكان المخصص)

أ- تحت 5 سنوات من العمر ( )

ب- 5 سنوات وما فوق ( )

11- هل لديك مرفق صحي في منطقتك؟

أ- نعم ( ) ب- لا ( )

اذا نعم،

12- ما هو نوع المرفق الصحي الذي تحصل فيه على خدمات الرعاية الصحية الخاصة بك؟

أ- نقطة غيار( ) ب - شفخانة ( )

ت- مركز صحي ( ) ث- وحدة الرعاية الصحية الأولية ( )

ج- مستشفى مرجعي ( ) ح - مستشفي طرفي ( )

و- القطاع الخاص ( ) ف- الذهاب مباشرة إلى الصيدلية ( )

ق - أخري ( ) حدد ....

13- ما هي المسافة التي يبعدها المرفق الصحي من منزلك؟

أ- أقل من 5كم ( ) ب- 5 كم ( )

ج- 5-10 كم ( ) د- أكثر من 10 كم ( ).

14- هل تتلقي الخدمة الصحية في المرفق الصحي الذي في منطقتك؟

أ- نعم ( ) ب - لا ( )

15- هل تحصل على الخدمة الصحية مجاناً؟

أ- نعم ( ) ب- لا ( )

إذا لا،

16- ما هى الطريقة التي تدفع بها في المرفق الصحي؟

أ- رسوم للخدمة ( ) ب- التأمين الصحي ( )

17- هل يمكن ان تتحمل دفع هذا المبلغ؟

أ- نعم ( ) ب- لا ( )

18- كم كنت تدفع كرسوم مقابلة الطبيب في كل مقابلة ؟

أ- أقل من 20 جنيه ( ) ب- 20 -50 جنيه ( )

ج-50 - 100جنية ( ) د- أكثر من 100 جنيه ( )

19- كم كنت تدفع للأدوية في كل مقابلة ؟

أ- أقل من 20 ج ب- 20 – 50 ج

ج-50 - 100جنية ( ) د- أكثر من 100 جنيه ( )

20 - كم كنت تدفع للفحوصات في كل مقابلة ؟

أ- أقل من 20 ج ب- 20 – 50 ج

ج-50 - 100جنية ( ) د- أكثر من 100 جنيه ( )

21 - ماهي تكلفة الحصول علي الخدمات الصحية في السنة؟..........................

22 - هل تتمكن من الحصول علي الخدمات الصحية بشكل منتظم ؟

أ- نعم ( ) ب- لا ( )

إذا (لا) جاوب علي السؤال322, اذا (نعم) اذهب ل 24

23 - ما هي العوامل التي تجعل الحصول عل الخدمة غير منتظم؟(يمكن إختيار أكثر من إجابة)
أ- الخدمات الصحية غير متوفرة ( ) ب- القيود المالية ( )

ج-عدم انتظام توافر الأدوية ( ) د- بعد المنشأه من منطقتك ( )
ت-عدم انتظام توافر الكوادر الصحية ( ) ث- عدم وجود الفحوصات المختبربة ( )

و-العاملين الصحيين غير مؤهلين ( ) ف- عدم وجود وسائل النقل ( )

ق- المعاملة السيئة من قبل العاملين في المجال الصحي ( ) ن- أخرى ( )

حدد ....

24- هل المرافق الصحية مفتوحة كلما تحتاج اليها؟
أ- نعم ( ) ب- لا ( )

25- إذا لم تكن الخدمات الصحية متوفرة في المرفق صحي القريب منك ماذا تفعل؟
أ- التحويل من قبل الكادر الصحي الي منشأه أخري ( ) ب- الذهاب لمرفق أخر مباشرة ( )
ج- استخدام علاج المنزلي ( ) د- الذهاب إلى معالج تقليدي ( )

ت- الحصول على العلاج من الصيدلية ( )

26 - هل الخدمات الصحية التي تتحصل عليها مناسبة لاحتياجاتك؟
أ- نعم ( ) ب- لا ( )

<u>جودة خدمات الرعاية الصحية:</u>

27- هل يمكن أن تصنف جودة الخدمات الصحية التي تحصل عليها الأن بعد لامركزية الخدمات الصحية؟
أ- متدهوره ( ) ب - تحسنت ()
28- هل العاملين في مجال الصحة متوفرين بانتظام في المرافق الصحية الخاصة بك؟
أ- نعم ( ) ب- لا ( )

ذا نعم،

29 - ما هوالمتاح من الكوادر الصحية في المرافق الصحية الخاصة بك؟ (يمكن أختيار اكثر من اجابة)

أ- المساعدين الطبين ( ) ب- الممرضين ( )

ج- الطبيب العمومي ( ) د- إختصاصيين ( )

ت- الدايات ( ) ث- أخري ( ) حدد............

30 - هل الأدوية متوفرة بشكل منتظم في مرفقك الصحي؟

أ- نعم ( ) ب- لا ( )

31 - هل تتلقي معلومات صحية(تثقيف صحي) في المرفق الصحي القريب منك؟

أ- نعم ( ) ب- لا ( )

32 - هل تثق في الموظفين الصحيين في المرفق الصحي؟

أ- نعم ( ) ب- لا ( )

33- هل تنتظر لفترة طويلة لتلقي الخدمات الصحية في المرفق الصحي؟

أ- نعم ( ) ب- لا ( )

34 - كم من الوقت تنتظر بالدقائق لتلقي الخدمات الصحية فى المرفق الصحي القريب منك ؟

....

35 - هل اختبرت أي تحسن في الحصول على الخدمات الصحية ما بعد اللامركزية؟

أ- نعم ( ) ب- لا ( )

36 - هل تكاليف العلاج أصبحت معقولة بعد اللامركزية؟

أ- نعم ( ) ب- لا ( )

37 - هل تشعربأنك مرحب بك في المرفق الصحي بعد اللامركزية؟

أ- نعم ( ) ب- لا ( )

38 - هل أنت راض عن نوعية الخدمات الصحية التي تحصل عليها؟

أ- نعم ( ) ب- لا ( ) ج- لا أعرف ( )

39 - ما هي نوعية مباني المرافق الصحية الان (بعد للامركزية)؟

أ- جيده ( ) ب- سيئة ( )

40- ما هو تقييمك لي مؤشرات جودة الخدمة بعد اللامركزية؟

#	المؤشر	تدهورت	تحسنت
1	توفر الأدوية		
2	توفر المرافق الصحية		
3	مسافة المرفق الصحي من البيت		
4	القدرة على تحمل تكاليف رسوم مقابلة الطبيب		
5	نوعية مباني المرافق الصحية		
6	القدرة على تحمل تكاليف الأدوية		
7	القدرة على تحمل تكاليف الفحوصات		
8	توفر الكوادر الصحيه		
9	توفر المعلومات ذات الصلة بالصحة		
