# Supplementary material for: Perceived consequences of healthcare service decentralization on access, affordability and quality of care in Khartoum locality, Sudan
Source: BMC Health Serv Res. 2021 Jun 17;21:581. doi: 10.1186/s12913-021-06479-0 (PMC8212465; doi:10.1186/s12913-021-06479-0)
Supplement: Supplementary file 3 — Additional file 3. English version interview guides for community members:This file captues the experienced change in the availability, affordability, accessbility and quality of health care services after implementation of decentralization as experienced by community members. [file 12913_2021_6479_MOESM3_ESM.pdf]

INTERVIEW THEMES FOR HEALTHCARE SERVICES USERS (community members)

Q1: What is your experience in seeking health service, or how you get health service when you or one of family member becomes ill?

Q2 How your experience has been changed after 2011(decentralization of health service)?

Q3: What are three important experienced positive changes that occurred after decentralization?

Q4: What are three important experienced negative changes that occurred after decentralization?

Q5: Did you experience any change in quality of health service after decentralization? and how?

Q6: Did you experience any change in the availability of health services and your accessibility to those services after decentralization

Q7: What are your suggestions to improve the implementation of decentralization?
