# Supplementary material for: Perceived consequences of healthcare service decentralization on access, affordability and quality of care in Khartoum locality, Sudan
Source: BMC Health Serv Res. 2021 Jun 17;21:581. doi: 10.1186/s12913-021-06479-0 (PMC8212465; doi:10.1186/s12913-021-06479-0)
Supplement: Supplementary file 4 — Additional file 4. Arabic version of interview guides for community members: This file captues the experienced change in the availability, affordability, accessbility and quality of health care services after implementation of decentralization as experienced by community members. [file 12913_2021_6479_MOESM4_ESM.pdf]

ARABIC HEALTH SERVICES USERS INTERVIEW THEMES

موضوعات المقابلة العميقة للمستخدمين

ما هي تجربتك في تلقي الخدمات الصحية، أو كيف تحصل على الخدمة الصحية عندما تكون أنت أو أحد أفراد الأسرة مريضا؟

كيف تغيرت تجربتك في تلقي الخدمات الصحية بعد عام 2011 (لامركزية الخدمات الصحية) ؟

ما هي أهم ثلاثة تغييرات إيجابية حدثت بعد اللامركزية؟

ما هي أهم ثلاثة تغييرات سلبية حدثت بعد اللامركزية؟

هل واجهت أي تغيير في نوعية الخدمات الصحية بعد اللامركزية؟ و كيف؟

هل واجهت أي تغيير في تواجد الخدمات الصحية ووصوليتك هذه الخدمات بعد تنفيذ لامركزية الخدمات الصحية؟

ما هي اقتراحاتكم لتحسين تنفيذ اللامركزية؟
