# Supplementary material for: Perceived consequences of healthcare service decentralization on access, affordability and quality of care in Khartoum locality, Sudan
Source: BMC Health Serv Res. 2021 Jun 17;21:581. doi: 10.1186/s12913-021-06479-0 (PMC8212465; doi:10.1186/s12913-021-06479-0)
Supplement: Supplementary file 5 — Additional file 5. Arabic version of health care providers interview guides: This file captures the multiple realities regarding the perception of health care providers about the change in working environment, quality of delivered services after the implementation of decentralization. [file 12913_2021_6479_MOESM5_ESM.pdf]

ARABIC HEALTH CARE PROVIDERS INTERVIEW THEMES

العمر:

الوظيفة:

كم من الزمن وانت تعمل في هذه الوظيفة:

1Q حدثنا عن تجربتك مع تطبيق لامركزية الخدمات الصحية؟

2Q من خلال تجربتك ماهي التغييرات التي طرأت بشأن بيئة العمل الخاصة بك (البنية التحتية والمستلزمات الطبية والأدوية) بعد تطبيق لامركزية الخدمات الصحية ؟

3Q هل تلقيت تدريب حول لامركزية الخدمات الصحية ؟

4Q :ما هي تصوراتك للتغيير بخصوص تقديم الخدمات الصحية للمرضى بعد لامركزية الخدمات الصحية ؟

5Q ما هي ثلاثة تغييرات إيجابية حدثت لامركزية الخدمات الصحية ؟

6 Q ما هي ثلاثة تغييرات سلبية حدثت لامركزية الخدمات الصحية ؟

7Q هل تشعر بأنك راض عن عملك؟ لماذا؟

8Qهل أنت أكثر انخراطا في اتخاذ القرارات في المسائل الإدارية لامركزية الخدمات الصحية ؟

9Q هل ميزانية المستشفى أكثر ملائمة بعد لامركزية الخدمات الصحية ؟

10Q ما هي اقتراحاتكم لتحسين تنفيذ لامركزية الخدمات الصحية ؟
