# Supplementary material for: Perceived consequences of healthcare service decentralization on access, affordability and quality of care in Khartoum locality, Sudan
Source: BMC Health Serv Res. 2021 Jun 17;21:581. doi: 10.1186/s12913-021-06479-0 (PMC8212465; doi:10.1186/s12913-021-06479-0)
Supplement: Supplementary file 6 — Additional file 6. English version of health care providers interview guides: This file captures the multiple realities regarding the perception of health care providers about the change in working environment, quality of delivered services after the implementation of decentralization. [file 12913_2021_6479_MOESM6_ESM.pdf]

INTERVIEW THEMES FOR HEALTHCARE PROVIDERS

Age

Occupation

For how long you are in this job

Q1: How you experience the process of decentralization implementation process?

Q2: what are experienced changes regarding your work environment (infrastructure, medical supplies and drugs) after decentralization?

Q3: Have received training about decentralization of health services?

Q4: What are three important experienced positive changes that occurred after decentralization?

Q5: What are three important experienced negative changes that occurred after decentralization?

Q6: What are your perceptions of change regarding provision of health service for patients after decentralization?

Q7: Do you feel satisfied about your job? Why?

Q8: Are you more involved in decisions in administrative matters after decentralization?

Q9: Is the budget for the hospital more adequate after decentralization?

Q10: What are your suggestions to improve the implementation of decentralization?
